# Adverse Drug Reactions in a Tertiary Care Emergency Medicine Ward - Prevalence, Preventability and Reporting

**DOI:** 10.1371/journal.pone.0162948

**Published:** 2016-09-13

**Authors:** Diana M. Rydberg, Lennart Holm, Ida Engqvist, Jessica Fryckstedt, Jonatan D. Lindh, Carl-Olav Stiller, Charlotte Asker-Hagelberg

**Affiliations:** 1 Clinical Pharmacology, Department of Medicine Solna, Karolinska Institutet, Stockholm, Sweden; 2 Clinical Pharmacology, Drug Safety and Evaluation Sector, Karolinska University Hospital Solna, Stockholm, Sweden; 3 Medical Product Agency, Uppsala, Sweden; 4 Department of Medicine, Department of Emergency Medicine, Karolinska University Hospital Solna, Karolinska Institutet, Stockholm, Sweden; 5 Division of Clinical Pharmacology, Department of Laboratory Medicine, Karolinska Institutet, Stockholm, Sweden; 6 Dept. of Clinical Pharmacology, Karolinska University Hospital Huddinge, Stockholm, Sweden; King Abdullah International Medical Research Center, SAUDI ARABIA

## Abstract

**Purpose:**

To identify the prevalence and preventability of adverse drug reactions (ADRs) in an emergency ward setting in a tertiary hospital in Sweden and to what extent the detected ADRs were reported to the Medical Product Agency (MPA).

**Methods:**

In this prospective cross sectional observational study, 706 patients admitted to one of the Emergency Wards, at the Karolinska University Hospital in Solna, Stockholm during September 2008 –September 2009, were included. The electronic patient records were reviewed for patients’ demographic parameters, prevalence of possible ADRs and assessment of their preventability. In addition, the extent of formal and required ADR reporting to national registers was studied.

**Results:**

Approximately 40 percent of the patient population had at least one possible ADR (n = 284). In the multivariable regression model, age and number of drugs were significantly associated with risk of presenting with an ADR (p<0.01 and p<0.001, respectively). Sex was not identified as a significant predictor of ADRs (p = 0.27). The most common ADRs were cardiovascular, followed by electrolyte disturbances, and hemorrhage. In 18 percent of the patient population ADRs were the reason for admission or had contributed to admission and 24% of these ADRs were assessed as preventable. The under-reporting of ADRs to the MPA was 99%.

**Conclusions:**

ADRs are common in Emergency Medicine in tertiary care in Sweden, but under-reporting of ADRs is substantial. The most frequent ADRs are caused by cardiovascular drugs, and significantly associated with age and number of drugs. However, only a minority of the detected serious ADRs contributing to admission could have been avoided by increased risk awareness.

## Introduction

Drug-related problems (DRP), including adverse drug reactions (ADRs), constitute a significant health- and quality problem particularly affecting the elderly [1]. Based on prevalence studies in different settings, approximately 5 to 35% of hospital admissions are due to adverse drug reactions (ADR) [2–8].

The definition of an ADR is harm directly caused by the drug at normal doses and during normal use compared to an adverse drug event (ADE) with a wider definition, including ADRs, overdoses, dose reductions and discontinuations of drug therapy [9]. The regulatory definition of an ADR is “an untoward and unintended harm by a drug independent of whether used within or outside the specifications of the medicinal product’s characteristics” [10]. In a small exploratory Swedish study, DRP including ADRs had either caused or contributed to almost one third of all admissions from the internal medicine emergency department in a tertiary care hospital [11]. According to a recent meta-analysis 1.6% of in-patients had preventable ADRs, and 45% of the ADRs were assessed as preventable [12]. Patient age, length of treatment with new drugs, total number of prescription drugs and hospital site (district general hospital vs regional teaching hospital) were variables associated with ADE admissions and preventability of ADEs [13]. In the general population, female gender has been identified as an independent risk factor for ADR-related hospital admissions by some [6, 14–18], but not all authors [19–25] analyzing different patient populations. In a Swedish study on elderly patients registered to receive home healthcare, 14% of hospital admissions were primarily caused by ADRs, with one-third of these ADRs related to impaired renal function, generally in very old women [26].

The most commonly reported preventable ADEs were related to inappropriate dosing and choice of: 1) antiplatelet drugs, anticoagulants, diuretics, angiotensin-converting enzyme inhibitors resulting in undesired cardiovascular reactions; 2) combinations of psychoactive agents, antiepileptic drugs causing central nervous system (CNS)–side effects; 3) opioids associated with respiratory depression; 4) anti-infective agents despite history of allergy [27].

According to EMA (European Medicines Agency) guidelines it is mandatory for health care professionals to report all suspected ADRs to the national competent authority. This feedback loop leads to improved safety information and may lead to increased pharmacovigilance with regard to certain side effects (Black box warning, black triangle) or contribute to withdrawal of market authorization.

The aim of this study was to determine the prevalence and preventability of ADRs in an emergency ward setting in a tertiary hospital in Sweden, considering age, the number of drugs (currently prescribed) and possible sex differences. In addition we assessed to which extent the detected ADRs were reported to the Medical Product Agency.

## Materials and Methods

### Study design

This prospective cross sectional observational study was a collaboration of the emergency department and the department of clinical pharmacology of Karolinska University Hospital Solna, one of seven emergency hospitals in Stockholm (2 million inhabitants), Sweden.

Following approval of the Regional Ethical Review Board (ERB) in Stockholm, Sweden (Etikprövningsnämnden, Dnr 2008/982-31/3 and 2009/2130-32), we included a sample of 706 adult patients (≥18 years) admitted to the Emergency Ward (AVA 1), via the emergency department ward between September 1, 2008 and September 31, 2009. During the months of September 2008, January 2009 and September 2009, all new admissions were included Monday to Friday. During a 12-week-period (October–December 2008), six new admissions were randomly included twice a week. The same population was also part of a larger study on drug related problems (DRPs) at the Emergency Ward within the same ethical approval (data not published).

At the time of the study (2008–2009) the emergency department had 80 000 annual visits and 2700 annual admissions to the Emergency Ward, AVA. The electronic patient records were reviewed and classified for possible DRPs by two emergency medicine physicians and two clinical pharmacologists (IE, JF, CAH, COS) and consensus was reached. The identified ADRs were reviewed in detail by a physician specialised in clinical pharmacology (DMR) with access to the electronic patient record. This review included ADR severity, causality and the preventability, as well as the contribution of the ADRs to admission. In addition, the impact of a decreased glomerular filtration rate (GFR) was assessed (JDL), using the CKD-EPI formula [28]. If needed, a registered nurse (RN) specialised in assessment and reporting of ADRs (LH) was consulted. The need for formal reporting of ADRs according to current pharmacovigilance legislation and records of ADR-diagnosis in the discharge letters was also noted.

### Classification of ADRs

The patient group with suspected ADRs did not include patients with intentional and self-inflicted intoxication since the legislation for formal reporting of ADRs at the time of the study did not include intentional and self-inflicted intoxication. Patients with suspected ADRs were classified using the Naranjo Score [29], based on the algorithm of the pharmacological characteristics of the suspicious drug, the dose–response relationship and a construction of causality of the ADRs, assigning the ADR to a probability category; definite, probable, possible, or doubtful. The medication most likely to cause or contribute to a particular ADR was registered by ATC-code classification [30]. If a patient presented with more than one ADR symptom, the clinically most important ADR was listed.

If an ADR-symptom was the main cause of the diagnosis the ADR was assessed as being the reason for admission. If an ADR aggravated the symptoms causing admission, the ADR was assessed as contributing to admission.

### Reporting ADRs to national authority

At the time of the study health, care professionals were obliged to report to the national authority (i) serious ADRs; (ii) ADRs not mentioned in the Summary of Product Characteristics (SPC); (iii) ADRs related to the use of new drugs (≤2 years after authorization) except those already labeled as common in the SPC; and (iv) ADRs that seem to be increasing in incidence. An important medical event, defined as not being immediately life-threatening or resulting in death or hospitalisation but may jeopardize the patient or may require intervention to prevent serious ADRs, should also usually be considered serious and be reported to the authorities [31]. We listed the adherence to the legal obligation to report ADRs to the national authority. Formal ADR-reports were retrieved from the national ADR registry held by the MPA.

### Serious ADRs

An ADR was assessed as serious if it fulfilled the World Health Organization (WHO) criteria for a serious adverse drug reaction, that is, if it was lethal, life-threatening, permanently disabling, lead to hospital admission, prolongation of hospital stay or classified as an “important medical event” [31, 32].

### Assessment of preventability

ADRs classified as causing or contributing to admission were selected for assessment of preventability according to Hallas’ avoidability criteria [33]. These include four categories, “definitely avoidable”, “possibly avoidable”, “not avoidable” and “unevaluable”.

### Statistical methods

The influence of patient characteristics on the probability of presenting with an ADR was investigated by means of multiple logistic regression, with age (as a continuous variable), number of drugs (root transformed due to skewness), and sex as independent variables. GFR was not included in the model due to pronounced collinearity with age. A p<0.05 was considered statistically significant. The association between sex, age and ADRs was analyzed by calculating the percentage of patients who presented with an ADR separately among women and men, in six age strata (≤24 years, 25–44 years, 45–64 years, 65–74 years, 75–84 years, and ≥85 years). The relative contribution of different drug classes to the overall risk of ADRs was investigated by calculating the number of suspected ADRs for each first-level (anatomical main group) ATC group, separately for women and men. For the ATC group most commonly associated with ADRs (C, cardiovascular system), the analysis was repeated at the second (therapeutic subgroup) ATC level. All statistical analyses were performed in R 3.0.2 (R Core Team (2013). R: A language and environment for statistical computing. R Foundation for Statistical Computing, Vienna, Austria. URL http://www.R-project.org).

## Results

### Patient population

The inclusion of the study patients was determined by day of admission in most cases and to a minor extent (18%) a random sample. In the total patient population (n = 706) women (n = 351) and men (n = 355) were evenly represented, women had a slightly higher median age than men. The ADR patient population (n = 284) was older with a higher percentage of women (54% vs 46%) compared to the non-ADR-population. The ADR-population also presented with a higher number of drugs, lower GFRs and longer duration of hospital stay (Table 1).

**Table 1:** Patient characteristics.

	All patients (n = 706)	Women (n = 351)	Men (n = 355)	ADR-patients (n = 284)	Non-ADR-patients (n = 422)
Age (years)	71 (58–82)	72 (59–84)	69 (57–81)	75 (63–84)	68 (53–81)
Number of drugs	6 (2–11)	7 (3–12)	6 (2–10)	8 (4–13)	5 (2–10)
Duration of hospital stay (days)	2 (2–4)	2 (2–4)	2 (2–3)	3 (2–4)	2 (2–3)
GFR (mL/min*)	72 (46–93)	72 (44–97)	71 (48–91)	65 (37–87)	75 (52–98)

All values are presented as median (inter quartile range),

*CKD-EPI formula

### ADRs

Approximately 40 percent of the whole patient population had at least one possible ADR (n = 284). In the multivariable regression model, age and number of drugs were significantly associated with the risk of presenting with an ADR (p<0.01 and p<0.001, respectively). Sex was not identified as a significant predictor of ADRs (p = 0.27). For unadjusted and confounder-adjusted estimates with their precision; see Table 2.

**Table 2:** Predictors for ADRs.

	Unadjusted OR^1^	Adjusted^3^ OR^1^
Age	1.023 (1.014; 1.032)	1.014 (1.004; 1.024)
Number of drugs^2^	1.50 (1.32; 1.71)	1.40 (1.22; 1.60)
Male sex	0.77 (0.57; 1.05)	0.84 (0.61; 1.15)

^1^ 0.95 Confidence Interval

^2^ root-transformed

^3^Model included age, number of drugs, and sex

**Fig 1:**
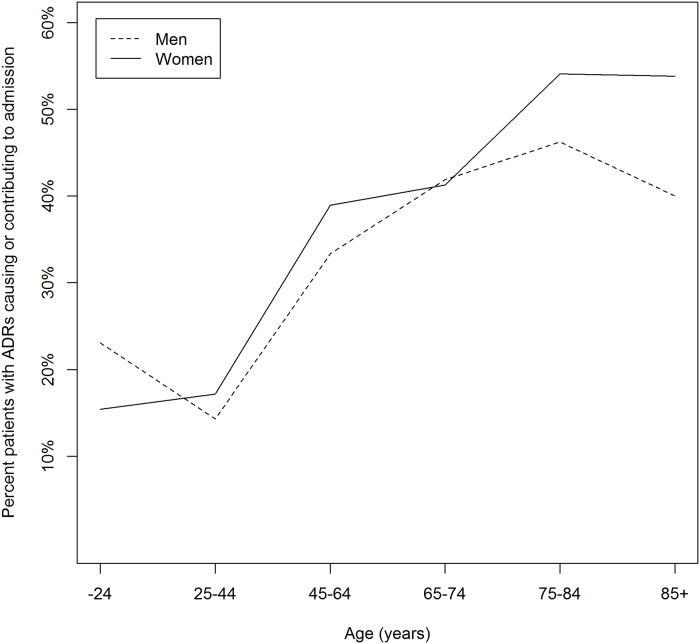
The distribution, in different age groups, of ADRs causing or contributing to admission, in women and men.

In 67 patients (10% of the study cohort) the ADR was the reason for admission and in 62 patients the ADR had contributed to admission. Although, sex was not significantly associated with the risk for ADRs per se, admission due to an ADR seemed to be more common in older women above 75 years as compared to men of that age group (Fig 1).

The most frequent ADRs (serious or non-serious) were cardiovascular ADRs, followed by electrolyte disturbances and hemorrhage. The medications responsible for cardiovascular ADRs were mainly antihypertensives; beta blocking agents, ACE-inhibitors and angiotensin receptor blockers (ARB). Electrolyte disturbances were caused by diuretics, ACE-inhibitors and ARB. Low-dose aspirin (acetylsalicylic acid, ATC-code B01) was the most common medication associated to hemorrhage (S1 Table).

Serious ADRs were detected in 138 patients and 76 (55%) of these patients were women. The most frequent serious ADRs were hemorrhages, followed by cardiovascular ADRs and blood dyscrasias (S2 Table).Serious hemorrhages were mainly caused by antithrombotic agents (n = 26). Blood dyscrasias such as febrile neutropenia were associated to antineoplastic agents. Serious cardiovascular ADRs (n = 36) were caused by beta blocking agents, agents acting on the renin-angiotensin system, psycholeptics (antipsychotics, anxiolytics, hypnotics, sedatives) and agents for cardiac therapy; digoxin (S2 Table). The above mentioned serious ADRs were also the most frequent ADRs to cause admission (S3 Table).

The most frequent preventable ADRs were cardiovascular ADRs, followed by electrolyte disturbances and hemorrhage, followed by ADRs affecting the central nervous system, CNS. The most common causative drugs were antihypertensive drugs and antithrombotic agents (S4 Table).

**Fig 2:**
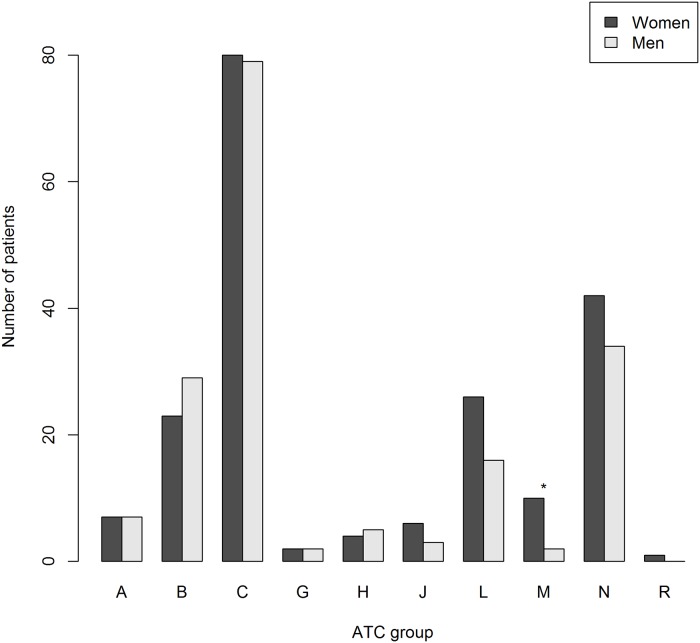
The distribution of suspected drugs causing ADRs in women and men, according to first level, anatomical main ATC group. Alimentary tract and metabolism (A), Blood and blood forming organs (B), Cardiovascular system (C), Reproductive system (G), Endocrine system (H), Infections (J), Antineoplastic and immunomodulating agents (L), Muscle, bones and joints (M), Brain and nervous system (N), Respiratory system (R). *p = 0.04

**Fig 3:**
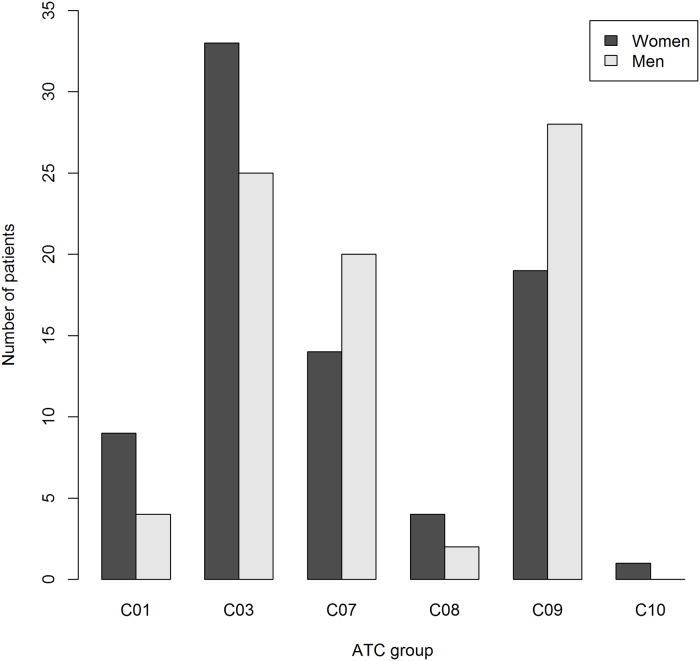
The distribution of suspected cardiovascular drugs causing ADRs in women and men, according to therapeutic subgroup (second level) of the ATC group. Cardiac therapy (C01), Diuretics (C03), Beta blocking agents (C07), Calcium channel blockers (C08), Agents acting on the renin-angiotensin system (C09), Lipid modifying agents (C10).

The majority of ADRs were linked to drugs commonly used for cardiovascular diseases, ATC-group B and C, as well as drugs affecting the nervous system (ATC-group N). Antineoplastic and immunomodulating agents (ATC-group L) were the fourth most common group (Fig 2). Medications in ATC-group M (e.g. NSAIDs) were significantly more common to cause ADRs in women compared to men (Fig 2). Among ATC group C drugs, “diuretics” (C03), “agents acting on the renin-angiotensin system” (C09) and “beta blocking agents” (C07) were the most common. There was a trend towards overrepresentation of diuretics in women and ACE-inhibitors/ARB in men (Fig 3).

### Assessment of ADRs

Sixty-nine percent (197/284) of the ADRs were assessed as “possible” and 29% (83/284) as “probable” according to Naranjo Score. Three ADRs (1%) were assessed as “definite”, two cases of increased drug levels of digoxin and lithium respectively and one case of hallucination caused by digoxin. Only one ADR (pruritus and acetylcysteine) was assessed as “doubtful”.

### Preventability of ADRs

Preventability assessment was restricted to ADRs causing or contributing to admission (n = 129). In this population, 24% (31/129) of the ADRs were considered “possibly avoidable” and all other ADRs were assessed as “not avoidable”. 18 percent of the ADRs were assessed as preventable in women (13/71) and 31% in men (18/58). An example of a “possibly avoidable” ADR was increased plasma levels of digoxin, leading to confusion, bradycardia and blurred vision in elderly patients with renal impairment.

### Formal reporting of ADRs

146 patients with ADRs (146/284) had at least one ADR which should have been reported to the Medical Products Agency according to the legislation at the time of the study. Only two reports were sent to the MPA. In the ADR patient population, there was a drug related diagnosis in the discharge letter in 6 percent (16/284) of the patients.

## Discussion

### Prevalence

The clinical setting and the high throughput of patients in the emergency ward makes it an ideal place to study the assumed prevalence of ADRs. The findings in this study indicate that ADRs are common in Emergency Medicine in tertiary care in Sweden. In 18 percent (129/706) of our cross-sectional sample of emergency ward patients ADRs had caused or contributed to admission. In the ADR population 45% (129/284) of the detected ADRs caused or contributed to admission. These results are in line with previous Swedish reports [7, 23], describing patients at a geriatric clinic or an internal medicine ward respectively.

A lower incidence of ADR-related hospital admission was presented in several meta-analyses not exclusively based on emergency care data [2–5, 34, 35]. When comparing the results from different studies, not only the patient population, but also the assessment of ADRs may differ, e.g. INR increased without hemorrhage, dizziness, thrombosis in cancer patients treated with antineoplastics and immunosuppressants may not be assessed as ADRs by all researchers.

In our study, age and number of drugs were risk factors for ADRs. GFR was not included in the model due to collinearity with age. GFR is known to decrease with advancing age, and it is possible that the association between age and ADRs to some extent could be accounted for by impaired renal function, leading to higher serum concentrations of renally excreted drugs. Indeed, in a post-hoc analysis, low GFR was significantly associated with the risk of ADRs (p<0.001, univariable regression), but not after adjusting for gender and number of drugs. Other studies have shown that patients admitted to hospital for ADRs were older and were taking a greater number of drugs than those admitted for other reasons [25, 36]. Our results also displayed the largest sex difference in admissions due to ADRs for the oldest patients 85+ (55% in women and 40% in men), which is in line with other studies showing a greater proportion of female patients admitted with ADRs than among patients hospitalized for other reasons [6, 15–18].

In our study, the most common drugs causing or contributing to admission were those with cardiovascular indications (i.e. diuretics, beta blocking agents, calcium channel blockers, ACE-inhibitors, ARBs and drugs for cardiac therapy) (29%), antineoplastic agents (17%) and antithrombotic agents (14%) (S3 Table). This pattern of causative agents may be explained by the relatively high number of patients treated at the oncology clinic (Radiumhemmet) and the thoracic clinic (Thoraxkliniken) of the Karolinska University Hospital Solna. According to a systematic review of prospective and retrospective studies, six different drug classes; (i.e. antibiotics, anticoagulants, digoxin, diuretics, hypoglycemics and NSAIDs), may account for 60–70% of all ADR-related hospital admissions [37].

### Preventability

Several previous studies have addressed the prevalence of ADRs in an emergency setting. Many ADRs could be prevented by avoiding certain drug combinations, anticipation of dose-dependent side effects, appropriate individual dosing considering renal function. In addition, patients should be educated to report signs of ADRs as soon as possible in order to avoid admission to the emergency ward.

Only approximately 1/4 of the ADRs causing or contributing to admission to the Emergency Ward, were assessed as preventable in this cross-sectional study. The focus of our study was the ADRs with the largest impact on patient health. We did not assess the preventability of all suspected ADRs present at the emergency department. Thus, our results may not be compared to the presence of all ADRs in a hospital population. According to two different reviews [27, 38] and a recent meta-analysis [12], the median preventability rate of all drug-related adverse events in inpatients was 35% and 46% (with a range between 19% and 90% in individual studies). The reasons for the wide range of preventability estimates in different studies may be attributable to different settings, assessment criteria and definitions of preventability [27].

In our study we found that antihypertensives such as diuretics, ACE-inhibitors and ARBs together with antithrombotics were the drugs causing the majority of the preventable ADRs (S4 Table). Admission to the emergency ward due to ADRs could have been prevented by: 1) better monitoring of cardiovascular drugs given in combination; 2) avoiding the combination of drugs with CNS side effects; 3) avoiding the combination of SSRIs and aspirin, at least in patients with high risk of hemorrhage; 4) dose adjustments of renally excreted drugs; 5) monitoring of electrolyte disturbances.

In an Irish study using the Hallas criteria, 5.3% of the ADRs were classified as “definitely avoidable”, 52% “possibly avoidable”, 33.3% unavoidable and 9.3% unclassifiable. The drugs causing dose-related and preventable ADRs were, similar to the findings in our study, cardiovascular drugs (diuretics, antithrombotics) and drugs affecting the CNS [19]. Also in line with our data, a systematic review found, drugs accountable for preventable drug-related hospital admissions to be aspirin (16%), diuretics (16%), NSAIDs (11%) and anticoagulants (8%) [39]. In a Swedish study on reported ADRs, a high number of preventable ADRs was found for CNS-active, cardiovascular and antithrombotic drugs, but not for cancer-related drugs [40].

### Reporting

Our study also confirms that ADRs to a large extent are underreported to the national authority, as only 1% (2/146) of the ADRs in this study was formally reported. This low reporting rate was seen when considering the legislation at the time of the study. The figure would have been even lower using the present legislation where all suspected ADRs, regardless of years on the market for the suspected causative drug or severity of the ADRs, should be formally reported. ADRs which cause admission are regarded as serious and are particularly important to report. The rate of spontaneous reporting of serious ADRs was 14% in a Swedish study investigating the reporting rate at five hospitals during a period of five years [41]. Attitudes to and incentives for reporting ADRs have been investigated in a sample of Swedish hospital physicians. The results show that hospital physicians regard severity of the reaction to be the most important factor for reporting and that under-reporting could be reduced if a web-based system for reporting was introduced [42].

Awareness and detection of ADRs in patients is important in tertiary care. This setting is ideal for optimizing patient safety with regards to drug use in general, and newly approved drugs in particular. Spontaneous reporting to competent authority is important pharmacovigilance signals that should not be delayed and reporting promptly is imperative. There may be several reasons for underreporting ADRs such as unawareness, lack of implemented routines and lack of time while on duty. Health care professionals awareness of the high prevalence of drug-related problems in general and adverse drug reactions in particular could be stimulated by continuous education and built in routines, reaching out also to the newly recruited doctors. Continuous on site clinical education and consultancy, including introduction of drug-focused routines and case based follow-up, by assistance from e.g. physicians specialised in clinical pharmacology should be considered. To facilitate the bureaucracy of ADR-reporting, e-reporting directed from the electronic patient record could also facilitate formal reporting. The large rate of underreporting of ADRs should stimulate a nationwide pharmacovigilance survey.

### Limitations of the study

Since the selection of patients was only randomized to a minor extent, we cannot exclude the possibility of selection bias. Furthermore, medical staff at the emergency ward was aware of this project which might have encouraged the reporting of suspected ADRs in the electronic patient record, indicating a higher prevalence of ADRs.

The classification and the assessment of the suspected ADRs causing or contributing to admission and the preventability were primarily assessed by a clinical pharmacologist consulting a specialised RN if needed. A higher stringency and perspective of the national authority assessing ADR might have been obtained if the RN had been consulted in all the assessments.

To facilitate the analyses of the assessments of the suspected ADRs, only one ADR was chosen for each patient, which underestimates the prevalence of ADRs to some extent. ADR-symptoms were classified according to established criteria. Most ADR-symptoms were categorized. However, some symptoms did not fit into the categories and we used the following assessments which may not be comparable to all other studies in this field. Although not associated with manifest hemorrhage, we chose to include “INR increased” as an ADR. A significantly increased INR is an imperative potential health hazard and will also often lead to extra considerations when treating a patient because of the elevated risk of bleeding. In one patient the increased INR was actually the single cause for admission and assessed as a preventable ADR. Another symptom commonly described by patients in the emergency ward, often causing or contributing to admission, is “dizziness”. In three out of five cases of dizziness assessed as ADR-symptoms in our study, the medication had either directly caused or contributed to admission.

To be classified as ADR “febrile neutropenia” the patient had to have a documented low leukocyte blood count. Patients with fever caused by antineoplastic and immunosuppressant drugs, but with normal leukocyte blood counts were classified as “pyrexia”. Three patients (patient 1; rituximab, patient 2; rituximab+methotrexate, patient 3; doxorubicin+docetaxel) classified as having “pyrexia” all had normal leukocyte counts in blood. In all three patients with “pyrexia” the ADR was the cause for admission.

Also the assessment of “thrombosis” as a possible ADR may seem difficult, since not only drugs but also certain diseases and immobility can cause this condition. If a study patient had used a drug known to increase the risk of thrombosis, and a contribution of the medication could not be ruled out thrombosis was assessed as a possible ADR.

In general, multiple chronic conditions are treated with a larger number of drugs. We did not analyze if the number of drugs which correlated to ADRs was due to comorbidity.

Extrapolation of prevalence of hospital admissions related to medication should be carried out with great caution because the prevalence strongly depends on setting and focus of the outcome. The prevalence of medication-related hospitalisations is lower in studies using other methods than medical chart review for the prevalence calculation [43].

In conclusion, ADRs are common in Emergency Medicine in tertiary care in Sweden and most frequently caused by cardiovascular drugs, and significantly associated with age and number of drugs. Under-reporting of ADRs is substantial. We encourage the physicians to report ADRs to a larger extent to the MPA. Instead of keeping record of all ADRs, the focus should be on those ADRs that could be prevented, in order to improve patient safety regarding drug treatment.

## Supporting Information

S1 Table All ADRs and suspected drugs. (DOCX)

S2 Table Serious ADRs and suspected drugs. (DOCX)

S3 Table ADRs causing or contributing to admission. (DOCX)

S4 Table Preventable ADRs. (DOCX)
