# Supplementary material for: Adverse Drug Reactions in a Tertiary Care Emergency Medicine Ward - Prevalence, Preventability and Reporting
Source: PLoS One. 2016 Sep 13;11(9):e0162948. doi: 10.1371/journal.pone.0162948 (PMC5021364; doi:10.1371/journal.pone.0162948)
Supplement: S4 Table — (DOCX) [file pone.0162948.s004.docx]

**S4 Table. Preventable ADRs**

|  | Cardiovascular ADRs* | Electrolyte disturbances* | Haemorrhage* | CNS ADRs* | Gastorintestinal ADRs* | Blood sugar disturbances* | Drug level increased | International normalised  ratio increased | Lactic acidosis | Renal impairment* | Total |
| --- | --- | --- | --- | --- | --- | --- | --- | --- | --- | --- | --- |
| C03 "DIURETICS" | 1 | 5 |  |  |  |  |  |  |  |  | 6 |
| C09 "AGENTS ACTING ON THE RENINANGIOTENSIN SYSTEM" | 3 | 1 |  |  |  |  |  |  |  |  | 4 |
| B01 "ANTITHROMBOTIC AGENTS" |  |  | 2 |  |  |  |  | 1 |  |  | 3 |
| C01 "CARDIAC THERAPY" | 1 |  |  | 1 |  |  | 1 |  |  |  | 3 |
| C07 "BETA BLOCKING AGENTS" | 3 |  |  |  |  |  |  |  |  |  | 3 |
| M01 "ANTIINFLAMMATORY AND ANTIRHEUMATIC PRODUCTS" |  |  | 2 |  |  |  |  |  |  | 1 | 3 |
| N02 "ANALGESICS" |  |  |  | 1 | 2 |  |  |  |  |  | 3 |
| N06 "PSYCHOANALEPTICS" | 1 | 1 | 1 |  |  |  |  |  |  |  | 3 |
| J01 "ANTIBACTERIALS FOR SYSTEMIC USE" |  |  |  | 2 |  |  |  |  |  |  | 2 |
| A10 "DRUGS USED IN DIABETES" |  |  |  |  |  | 1 |  |  | 1 |  | 1 |
| A11 "VITAMINS" |  | 1 |  |  |  |  |  |  |  |  | 1 |
| A12 "MINERAL SUPPLEMENTS" |  | 1 |  |  |  |  |  |  |  |  | 1 |
| C08 "CALCIUM CHANNEL BLOCKERS" | 1 |  |  |  |  |  |  |  |  |  | 1 |
| G04 "UROLOGICALS" | 1 |  |  |  |  |  |  |  |  |  | 1 |
| H01 "PITUITARY AND HYPOTHALAMIC HORMONES AND ANALOGUES" |  | 1 |  |  |  |  |  |  |  |  | 1 |
| H02 "CORTICOSTEROIDS FOR SYSTEMIC USE" |  |  | 1 |  |  |  |  |  |  |  | 1 |
| M05 "DRUGS FOR TREATMENT OF BONE DISEASES" |  |  |  |  | 1 |  |  |  |  |  | 1 |
| Total | 11 | 10 | 6 | 4 | 3 | 1 | 1 | 1 | 1 | 1 |  |

*Clinical manifestations of ADRs included in the categories above

**Cardiovascular ADRs**: “arrhythmia”, “cardiac failure”, “hypertension”, “hypotension”, “orthostatic hypotension”, “syncope”

**Electrolyte disturbances**: “hypercalcemia”, “hyperkalemia”, “hypokalemia”, “hyponatremia”

**Hemorrhage:** “epistaxis”, “gastric ulcer”, “gastrointestinal hemorrhage”, “hematemesis”, “hematoma”, “hematuria”

**CNS ADRs:** “confusional state”, “depressed level of consciousness”, “hallucination”, “somnolence”, “respiratory depression”, “parkinsonism”, “fatigue” , ”headache”

**Gastrointestinal ADRs:** “constipation”, “diarrhea”, “gastritis”, “vomiting”, “nausea”

**Blood sugar disturbances:** “diabetes mellitus”, “hyperglycemia”, “hypoglycemia”

**Renal impairment:** ”blood creatinine increased”, ”acute renal failure”
